# Development of Pig Conventional Dendritic Cells From Bone Marrow Hematopoietic Cells *in vitro*

**DOI:** 10.3389/fimmu.2020.553859

**Published:** 2020-10-08

**Authors:** Yanli Li, Lucinda Puebla-Clark, Jesús Hernández, Ivan Díaz, Enric Mateu

**Affiliations:** ^1^Departament de Sanitat i Anatomia Animals, Facultat de Veterinària, Universitat Autònoma de Barcelona, Barcelona, Spain; ^2^Laboratorio de Inmunología, Centro de Investigación en Alimentación y Desarrollo, Hermosillo, Mexico; ^3^Centre de Recerca en Sanitat Animal, IRTA-UAB, Bellaterra, Spain

**Keywords:** Flt3L, cDC1, cDC2, CD14^+^ DC, pig

## Abstract

In recent years, porcine dendritic cells (DCs) have been identified from pig tissues. However, studying the interaction of porcine DCs with pathogens is still difficult due to the scarcity of DCs in tissues. In the present work, the Flt3-ligand (Flt3L)-based *in vitro* derivation system was further characterized and compared with other cytokine derivation models using a combination of factors: stem cell factor (SCF), GM-CSF, and IL-4. The method using Flt3L alone or combined with SCF supported the development of pig bone marrow hematopoietic cells into *in vivo* equivalent conventional DCs (cDCs). The equivalent cDC1 (the minor population in the cultures) were characterized as CADM1^+^CD14^–^MHC-II^+^CD172a^–/*lo*^CD1^–^CD163^–^ DEC205^+^CD11R3^*lo*^CD11R1^+^CD33^+^CD80/86^+^. They expressed high levels of FLT3, ZBTB46, XCR1, and IRF8 mRNA, were efficient in endocytosing dextran and in proliferating allogenic CD4^+^CD8^+^ T cells, but were deficient in phagocyting inactivated *Staphylococcus aureus* (*S. aureus*). Also, after poly I:C stimulation, they predominantly produced IL-12p40a and matured as indicated by the increase of MHC-I, MHC-II, and CD80/86. The equivalent cDC2 (the main population) were CADM1^+^CD14^–^MHC-II^+^C D172a^+^CD1^+^CD163^–/*lo*^DEC205^*lo*^CD11R3^+^CD11R1^+^CD33^+^CD80/86^+^; meanwhile, they overexpressed FcεR1α and IRF4 mRNA. They showed high efficiency in the endocytosis of dextran, but weak in phagocytosing bacteria. They supported allogenic CD4^+^CD8^–^/CD4^+^CD8^+^ T cell proliferation and were high producers of IL-12p40 (upon TLR7 stimulation) and IL-10 (upon TLR7 stimulation). TLR ligand stimulation also induced their maturation. In addition, a CD14^+^ population was identified with the phenotype CADM1^+^CD14^+^MHC-II^+^CD172a^+^ CD1^+^CD163^+^DEC205^–^CD11R3^+^CD11R1^+^CD33^–/*lo*^CD80/86^+^. They shared some functional similarities with cDC2 and were distinguishable from macrophages. This CD14^+^ population was efficient in phagocyting *S. aureus* but showed less maturation upon TLR ligand stimulation than cDC1 or cDC2. The alternative methods of DC derivation including GM-CSF and/or IL-4 produced mostly CADM1^–^ cells that did not fulfill the canonical phenotype of *bona fide* porcine DCs. Our study provides an exhaustive characterization of Flt3L-derived DCs with different methods that can help the *in vitro* study of the interaction of DCs with porcine-relevant pathogens.

## Introduction

Pig farming is an important source of animal protein. As of January 2020, it was estimated that about 677 million pigs were raised worldwide and are therefore an important source of economic income in many countries (1). Additionally, pigs are epidemiologically important hosts of pathogens that can affect humans such as the H1N1 “swine flu” pandemic in 2009 (2). Pigs are also models for studying several human diseases (3) and potential donors for xenotransplantation (4). In all those aspects, a better understanding of the pig immune system and of the interaction of pathogens with it is scientifically relevant.

Dendritic cells (DCs) are sentinels of the immune system, essential for the activation of naïve T cells to promote the initiation of the adaptive immune response. Currently, there are different types of DCs that are recognized based on their ontogeny (5, 6): the monocyte-derived DCs (moDCs), which are generated from activated monocytes in the tissues (7), and the conventional or *bona fide* DCs that are generated from Flt3 (CD135)-expressing precursors (8) and can be subdivided into classical/conventional (c) cDC1 and cDC2, which can be differentiated by gene expression and cytokine profiles (5, 6) and their ability to cross-present antigens (9, 10). Additionally, there are plasmacytoid DCs (pDCs), specialized in producing high amounts of type I interferons (especially IFN-α) during antiviral responses (11, 12), and finally, Langerhans cells are a special type of DCs generated from erythro-myeloid progenitors (6).

One limitation when studying *bona fide* DCs in pigs or other species is the low frequency of these cells. For example, in pig blood, cDC1, cDC2, and pDC accounted for only 0.1, 0.1, and 0.5-1%, respectively, of the intact Peripheral Blood Mononuclear Cells (PBMCs) (13); in lung, 5.6 ± 5.1% and 11.8 ± 12.3% of the MHC-II^*hi*^ cells were identified as potential cDC1 and cDC2, respectively (14); in lymphoid tissues, 56.9 ± 3.1% and 30.1 ± 8.5% of cells were identified as cDC1 and cDC2, respectively, among lymphocyte-depleted MHC-II^*hi*^CADM1^*hi*^ cells (15). This scarcity makes it extremely difficult to perform functional analysis or to investigate DC functionality upon sensing pathogens. Apart from that, when obtaining DCs from tissues, the extraction process imposes a series of manipulation that may damage cell surface receptors or modify cell maturation status, which subsequently will deviate the DC functionality from *in vivo* conditions. Thus, *in vitro* production of DCs can be a convenient alternative. A review of the literature shows that, for many years, production of DCs in pigs was done by stimulating monocytes with GM-CSF and IL-4 or bone marrow precursors with GM-CSF (16). While the first method consistently produced CD163^+^ moDCs, the second generated a more heterogeneous population (16).

In human and mice, *bona fide* DCs are identified as cells originating from a common macrophage and DC precursor (MDP), which gives rise to a common DC precursor (CDP), restricted to develop into cDCs (cDC1 and cDC2), and pDC (6, 17). Flt3 seems to be a key molecule in DC development. In mice, Flt3 is broadly expressed on hematopoietic precursors and exists throughout the whole process of DC development (18) and during commitment to DCs; the expansion and migration of precursor cells are essentially Flt3L-dependent processes (8). In pigs, Guzylack-Piriou et al. (19) showed that addition of Flt3 ligand (Flt3L) to bone marrow cell cultures produced cells with features of *bona fide* DCs. In the past years, DCs from pig lymphoid (spleen, tonsil, and lymph nodes) (15, 20) and non-lymphoid organs (skin and lung) (14, 21) as well as blood (22) have been successfully identified and characterized. From the abovementioned studies, consensus has been reached on the phenotype of cDC1 (CADM1^+^CD14^–^MHC-II^+^CD172a^–/*lo*^), cDC2 (CADM1^+^CD14^–^MHC-II^+^CD172a^+^), and pDC (CADM1^–^CD14^–^CD172a^+^CD4^+^). In the present study, we re-examined the features of pig DCs produced in an Flt3L-dependent culture model regarding the phenotype, gene expression, and functionality. Moreover, we compared the use of FLt3L alone with other cytokine combination [addition of stem cell factor (SCF), GM-CSF, and IL-4] in order to determine the effects on the yield and type of DCs produced.

## Materials and Methods

### Obtention of Bone Marrow Cells

Porcine bone marrow hematopoietic cells (BMHCs) were aseptically obtained from femora and humeri from 4-week-old pigs as previously described (16). Animals were all Landrace × Large White and came from the same source farm. BMHCs were tested by PCR to confirm that they were free of *porcine reproductive and respiratory syndrome virus* (PRRSV; VetMAX PRRSV NA & EU Reagents; Thermo Fisher Scientific, Spain), *porcine circovirus type* 2 (PCV2), *Mycoplasma hyopneumoniae*, and *torque teno sus virus* (TTSuV) 1 and 2 (23–25). Cells were frozen in liquid nitrogen until used.

### Generation of DCs From Flt3L-Dependent Bone Marrow Cultures

For DC generation, BMHCs were seeded in 24-well plates at a density of 1 × 10^6^ cells/600 μl in RPMI 1640 medium supplemented with 10% fetal calf serum (FCS), 2 mM glutamine, 20 mM HEPES, 100 units/ml penicillin, and 100 μg/ml streptomycin. 20 nanograms per milliliter of recombinant human Flt3L (rhuFlt3L, Fisher Scientific, Spain) was added to promote expansion and differentiation of Flt3^+^ or relevant cells. Cells were incubated for 14 days at 37°C in a 5% CO_2_ and 90% humidity environment. Half of the medium was replaced every 3 days with fresh supplemented medium containing the same concentration of Flt3L.

To assess the development of DCs, cultures were examined at regular intervals during the 2-week period of incubation. For this purpose, BMHCs were labeled with 5 μM CellTrace Violet (Fisher Scientific, Spain), washed, and seeded at 0.8 × 10^6^ cells/600 μl into the 24-well plates. Proliferation was assessed at 0, 1.5, 3, 6, 9, 12, and 14 days of incubation by flow cytometry. Proliferated CADM1^+^MHC-II^+^ cells were considered as potential Flt3L-derived DCs. All experiments in the present work were done at least in triplicate except when indicated.

### Obtention of Alveolar Macrophages, Isolation of Peripheral Blood Mononuclear Cells and Monocytes, and Generation of Monocyte-Derived DCs

Alveolar Macrophages (AMs) and PBMCs were obtained from the same pigs as described in “Obtention of Bone Marrow Cells” through, respectively, broncho-alveolar lavage and density gradient centrifugation with Histopaque 1.077 (Sigma-Aldrich, Spain). AMs and PBMCs were frozen in liquid nitrogen until used. The selected batches had viabilities >90% after thawing as assessed by trypan blue staining. For PBMCs, the proportion of each T cell subset and the proliferation capability were also tested in one sample per batch. Batches passing this quality control (viability and proliferation capability) were used. Monocytes were obtained through plastic adherence of PBMCs for 16 h at 37°C in 5% CO_2_ and used freshly. moDCs were generated by differentiating blood monocytes with recombinant porcine GM-CSF (rpGM-CSF, 20 ng/ml; R&D Systems, Spain) and rpIL-4 (20 ng/ml; R&D Systems, Spain) according to the method described by Carrasco et al. (16).

### Flow Cytometry Analysis

Staining of cell surface molecules was performed in PBS with 5% FCS and 5% horse serum for 30 min on ice. Primary antibodies (conjugated or not) and their working dilutions are listed in Table 1. When necessary, secondary antibodies, anti-mouse IgG1, IgG2a, and IgG2b coupled to Brilliant Violet 421 (BV421), Alexa Fluor 488, R-phycoerythrin (R-PE), or Alexa Fluor 647, were used at a dilution of 1:800 to 1:1600. The antibodies included in the panel were previously titrated to determine the appropriate dilution. Between each labeling step, cells were washed twice with PBS containing 2% FCS and centrifuged at 500 × *g* for 5 min. Samples were acquired on a MACSQuant Analyzer 10 (Miltenyi Biotec, Bergisch Gladbach, Germany) or sorted on BD FACSJazz (BD Biosciences, Oxford, United Kingdom). Fluorescence minus one (FMO) controls, matched isotype controls for mouse IgG1, IgG2a, and IgG2b, and background from secondary antibodies were used for analysis and gating. The acquired data were analyzed using FCS Express 7 (*de novo* Software, Glendale, CA, United States).

**TABLE 1:** Antibodies used for flow cytometry analysis.

Antibody	Clone	Isotype	Species produced	Working dilution	Supplier
Anti-CD14 FITC conjugated	MIL2	IgG2b	Mouse	1/100	Bio-Rad
Anti-CD172a	BL1H7	IgG1	Mouse	1/500	
Anti-SLA II DR	2E9/13	IgG2b	Mouse	1/500	
Anti-CD11R1	MIL4	IgG1	Mouse	1/500	
Anti-CD11R3	2F4/11	IgG1	Mouse	1/500	
Anti-CD1	76-7-4	IgG2a	Mouse	1/50	
Anti-CD163	2A10/11	IgG1	Mouse	1/250	
Anti-CD3	PPT3	IgG1	Mouse	1/200	
Anti-CD4a FITC conjugated	74-12-4	IgG2b	Mouse	1/200	BD Pharmingen
Anti-CD8a Alexa Fluor 647 conjugated	76-2-11	IgG2a	Mouse	1/100	
Anti-human CADM1	3E1	IgY	Chicken	1/1000	MBL
Anti-mouse CD80 (B7-1) Super Bright 436 conjugated	16-10A1	IgG2a	Armenian hamster	1/150	eBioscience
human CD152 (CTLA-4)-muIg fusion protein	–	IgG2a	Mouse	1/50	Ancell
Anti-DEC-205 (hybridoma supernatant)	9HZF7	IgG1	Mouse	1/1	Provided by Lab. de Inmunología, CIAD, A.C.
Anti-CD33 (Siglec-3) (hybridoma supernatant)	5D5	IgG1	Mouse	1/1	Gift from Dr. J. Domiìnguez*
Anti-MHC-I (hybridoma supernatant)	4B7/8	IgG2a	Mouse	1/1	

*Department of Biotechnology, INIA, Madrid, Spain. Antibodies without specific indications are anti-pig antibodies.

For phenotypic characterization of the generated DCs, a five-color staining was performed for cell surface molecules CADM1, CD14, MHC-II, and CD172a, plus a fifth marker targeting CD1/CD163/DEC205/CD11R3/CD11R1/CD33/CD80/86. Anti-CD11R3 and anti-CD11R1 were used to stain CD11b. These two antibodies were reported to be specific for CD11b but with different staining patterns in pigs (26). Cells were initially incubated with antibody anti-CADM1 followed by anti-chicken IgY biotin and the primary antibody of the fifth marker. Then, streptavidin PerCP-Cy5.5 and a secondary antibody coupled to BV421 were added. Finally, cells were stained with anti-CD14 FITC, anti-MHC-II conjugated to anti-mouse IgG2b Zenon Alexa Fluor 647 (Thermo Fisher Scientific, Spain), and anti-CD172a conjugated to LYNX Rapid RPE (Bio-Rad, Spain). For the combination with CD1, CD172a and CD1 were both indirectly labeled with secondary antibodies coupled to BV421 and RPE, respectively. CD80/86 was labeled by an antibody anti-mouse CD80 (B7-1) Super Bright 436.

### RNA Extraction and Quantitative Real-Time PCR

Total RNA from FACS-sorted DC subpopulations was extracted with TRIzol (Thermo Fisher Scientific, Spain) according to the manufacturer’s protocol and quantified with a Biodrop spectrophotometer (BioDrop, United Kingdom). Genomic DNA was removed using a gDNA removal kit (ArcticZymes, Sweden). Then, 20 ng was used to amplify mRNA transcripts of FLT3, ZBTB46, XCR1, BATF3, IRF4, IRF8, FCεR1a, ITGAM (CD11b), ITGAX (CD11c), CD64, CSF1R, MAFB, MERTK, KLF4, and MGL2 genes using iTaq universal SYBR green one-step kit (Bio-Rad, Spain) and the adequate primers (Table 2) in a 7500 Real-Time PCR System (Applied Biosystems). The same pig origin of AMs, monocytes, or GM-CSF/IL-4-differentiated moDCs were included for comparison as needed. Relative levels of mRNA were assessed according to the ΔCt method and calculated with the formula 2^–Δ^ ^*Ct*^. Both *Glyceraldehyde 3-phosphate dehydrogenase* (*GAPDH*) and *ribosomal protein 24* (*RSP24*) were used as reference genes as previously described (14). No significant difference was found when applying the normalization to the two reference genes; thus, the result to GAPDH was shown.

**TABLE 2:** Primers used for RT-qPCR.

Target mRNA	Primer sequences (5′–3′)	Gene ID	Product size (bp)	References
RPS24	F: CTTCAGCGGAAACAAATGGT R: CCTGTGTTTGGGCTCATTTT	100155012	219	Designed
GAPDH	F: CACCATCTTCCAGGAGCGAG R: AGAAGGGGCAGAGATGATGA	396823	154	(21)
FLT3	F: CAACTGCACGGAAGAGATCA R: GCACAGCACTTCACCAGAAA	100515445	132	Designed
XCR1	F: ACTGCTGGAAGAGGCTGTGT R: ATAAATGCCGGCAGAAGATG	414375	220	Designed
FCeR1α	F: ATTTACAGACCCACAGCCTAGCT R: TGCCAGTTACAGTTGGTTCAA	100152827	183	(21)
ITGAM (CD11b)	F: AGAAGGAGACACCCAGAGCA R: GTAGGACAATGGGCGTCACT	397459	197	(56)
ITGAX (CD11c)	F: GCTCCTTCGAGTTGGAGATG R: ACCAGGCTCTGTACCCCTTT	100738907	229	Designed
IRF4	F: CCAAAAGCAAAGATGGCATT R: ACCTGGAGCTCTCATGCTGT	100144625	181	Designed
IRF8	F: TCCTCCTTCTGAATCCGAACC R: GAGCAGGACTTGAGCGGAAA	396645	202	Designed
BATF3	F: GAGGAGCTGAAGCACCTGAG R: CGAAGCTCTGAGGAGGTGAC	100621683	249	Designed
ZBTB46	F: TGTCTCTCCTTTGGCTCGTT R: GACGGAATGTCAGTGTCACG	110257361	182	Designed
CD64 (FCGR1A)	F: CCACCAACTCCTGTCTGGTT R: TTACCTTCTTCCCGTGACCA	613130	155	Designed
CSF1R	F: CAGCAAGGACACGTACAGAT R: GGGTACAGAAGGGTGAGTTC	100517086	138	Designed
MAFB	F: GACTCCTGGCTTCCTGAACT R: TTCTCCGGACCCTAGACAGA	100518227	186	Designed
MERTK	F: GGACACAGGACCAAAGCATA R: ATCGCCGCTGTAAATCTTCT	100519652	193	Designed
CX3CR1	F: GGGAGGGAAAGGGAAATACTC R: GAAGCCCATCGTAGTCAAAGT	100622126	157	Designed
KLF4	F: AGTTCTCATCTCAAGGCACA R: CCAGTCACAGTGGTAAGGTT	595111	63	Designed
MGL2	F: ACTTCTCTGGAGAGCACACT R: GCTGTTGTACATGGTCGGTT	216864	82	Designed

### Dextran-FITC Uptake

Sorted DC subpopulations (with 4000 to 6000 cells used in each test) were incubated with Dextran-FITC (molecular weight 40,000; Sigma-Aldrich, Spain) at 1 mg/ml in complete RPMI 1640 for 2.5 h either at 37°C or on ice (negative control). Free Dextran-FITC was washed away, and uptake activity at both temperatures was determined by flow cytometry. The median fluorescence intensity (MFI) of cells incubated at 37°C was compared with the corresponding cells incubated on ice.

### Phagocytosis of *Staphylococcus aureus*

Sorted DC subpopulations (10,000 cells in each test) were incubated with Alexa 488-labeled *S. aureus* (Thermo Fisher Scientific, Spain) at 20 μg/ml (equivalent to a ratio of 60 particles per cell) in complete RPMI 1640 for 1 h either at 37°C or on ice (negative control). Extracellular bound *S. aureus* were quenched by addition of 1 mg/ml trypan blue before the analysis by flow cytometry.

### Mixed Lymphocyte Reaction Assay

To test antigenic presentation capabilities of each DC subpopulation, allogeneic PBMCs were used as a source of responder cells. These PBMCs were obtained from 12-week-old pigs (free of PRRSV, PCV2, Mycoplasmas, TTSuV 1 and 2, and influenza A) and then frozen and tested as described above. Then, they were labeled with CellTrace Violet (Fisher Scientific, Spain) and re-suspended in medium containing 10% FCS at 1 × 10^6^ cells/ml with 100 μl/well plated in the 96-well U-bottom plate. The sorted DC subpopulations (cDC1, cDC2, and CD14^+^ DCs) were added as stimulators at a ratio of 1:5 based on the number of allogeneic PBMCs. After 5 days of culture, cells were harvested and stained with anti-CD3, anti-CD4, and anti-CD8a antibodies (see Table 1 for details) before the flow cytometry analysis. PBMCs without DCs added were used as the negative control; AMs and monocytes (the same pig origin as the sorted DCs) were obtained as described above and used as other cell-type references. Results were reported as in previous studies (14) using a relative proliferation index where a value of 1 was assigned to the cell population producing the highest percentage of proliferating cells; all other subsets were normalized to it.

### Response to TLR Agonist Stimulation: Maturation Markers and Cytokine Patterns

Sorted DC subpopulations (cDC1, cDC2, and CD14^+^ DCs) were re-suspended at a density of 250,000 cells/ml with medium containing 10% FCS and were then seeded in 96-well U-bottom plates (200 μl/well, equivalent as 50,000 cells/well) in the presence of TLR agonists: 10 μg/ml poly I:C, 1 μg/ml LPS, or 10 μg/ml gardiquimod (all from InVivogen, reconstituted according to the manufacturer’s instructions). In parallel, control cells were cultured without the TLR agonists, and AMs were included as other cell-type reference. After 18 h of incubation, cells were stained for MHC-I, MHC-II, and CD80/86. For CD80/86 staining, human CD152 (CTLA-4)-muIg (Ancell, Bayport, MN, United States), which binds to CD80/86, was used (see Table 1). Supernatants were harvested and frozen at −80°C until used.

Presence of IFN-α, IFN-γ, IL-4, IL-6, IL-12p40, IL-10, IL-1β, TNF-α, and IL-8 (CXCL8) in the cell culture supernatants was measured by means of the Porcine ProcartaPlex Multiplex Immunoassay (Labclinics, Spain) according to the manufacturer’s instructions. Briefly, 50 μl of each sample was added to a well of a 96-well plate containing fluorescent antibody-coated beads. After overnight incubation, 25 μl of biotinylated detection antibodies followed by 50 μl of streptavidin-PE antibodies were added. Finally, beads in each well were re-suspended with 120 μl of assay buffer and analyzed by a MAGPIX system. Concentration of cytokines in each sample was determined by using standard curves, which were fitted by a four-parameter logistic regression model. All samples were run in duplicate.

### Effect of SCF, GM-CSF, and IL-4 Addition to Flt3L-Based BMHC Cultures

In subsequent experiments, additional cytokines that regulate DC lineage commitment and differentiation were added to compare with the use of Flt3L alone. SCF (Kingfisher Biotech, Spain) was added to test its effect on the expansion and survival of progenitor cells, which afterward was combined with the addition of GM-CSF (20 ng/ml; R&D Systems, Spain) or IL-4 (20 ng/ml; R&D Systems, Spain). The combination of cytokine cocktails included the following: (1) Flt3L (as standard method), (2) Flt3L + SCF, (3) Flt3L + SCF + GM-CSF (FSG), and (4) Flt3L + SCF + GM-CSF + IL-4 (FSG4). In cases (3) and (4), a two-step protocol was used. In the first step, BMHCs were cultured in the presence of Flt3L (either 20 or 100 ng/ml) and SCF (either 20 or 100 ng/ml) to expand progenitors. After 6 days of culture with one medium changed at day 3, cells were re-plated in cultures containing cytokine cocktails Flt3L + SCF + GM-CSF (FSG) or Flt3L + SCF + GM-CSF + IL-4 (FSG4) to induce further cell differentiation. Cells were kept for an additional 12-day period (18-day period in total) with cocktail replenishment after 6 days. All the cases were phenotypically analyzed by a four-color staining of CADM1, CD14, MHC-II, and CD172a as described under “flow cytometry analysis.”

### Statistical Analysis

All data were analyzed using the GraphPad Prism 8.0 software package (GraphPad Software, La Jolla, CA, United States). Statistical tests applied to each data set are indicated in the relevant figure legend.

## Results

### Three Distinct Cell Populations Can Be Identified in Flt3L-Derived Bone Marrow Cell Cultures

**FIGURE 1:**
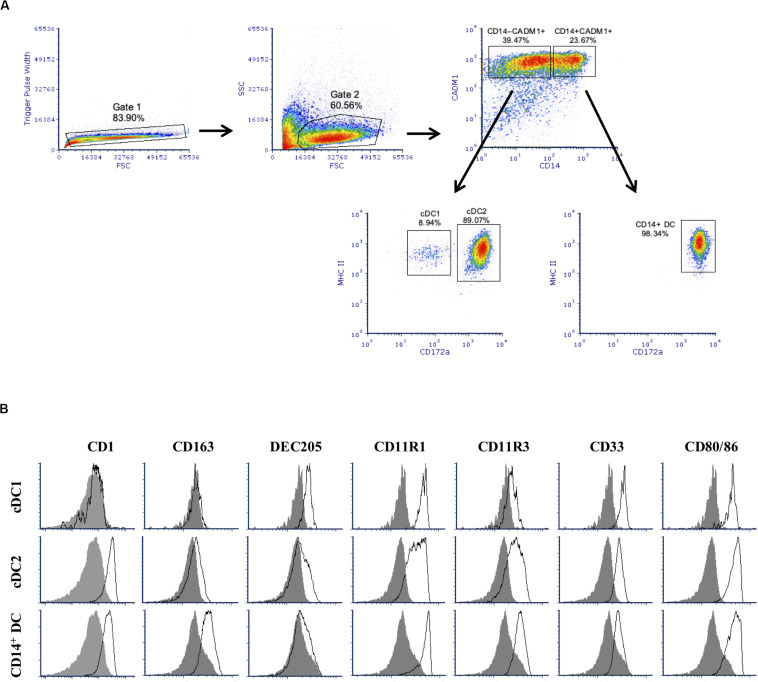
Phenotype of Flt3 ligand-derived dendritic cells (Flt3L-DCs). Cells were stained for multi-color flow cytometry. **(A)** Illustrative density plots show the gating strategy: singlets → excluding cell debris → selecting CADM1^+^ population and divided by CD14 → identify cDC1-putative and cDC2-putative populations based on MHC-II^+^ and different expression of cD172a. Three putative populations were identified with cDC1-putative population gated as CADM1^+^CD14^–^ MHC-II^+^CD172a^– /*lo*^, cDC2-putative population as CADM1^+^CD14^–^ MHC-II^+^CD172a^+^, and plus a CD14^+^ population (CADM1^+^CD14^+^MHC-II^+^CD172a^+^) without classification. **(B)** Expression of phenotypic markers CD1, CD163, DEC205, CD11R1, CD11R3, CD33, and CD80/86 on the defined cDC1-putative, cDC1-putative, and CD14^+^ populations was assessed by flow cytometry. The filled histogram represents the FMO control. The density plots and histograms shown are illustrative for one but represent four pigs.

After 2 weeks of culture, stimulation of BMHCs with Flt3L resulted in the development of a large subset of cells expressing CADM1 (59.5 ± 12.9%), among cells after gating singlets and excluding cell debris as shown in Figure 1A. Among those CADM1^+^ cells, three subpopulations could be identified based on the cell surface expression of CD14, MHC-II, and CD172a. The major one (52.9 ± 4.0%, among cells of CADM1^+^) presented a phenotype of CD14^–^MHC-II^*hi*^CD172a^+^ that was compatible with cDC2. The second population was CD14^–^MHC-II^*hi*^CD172a^–/*lo*^, which represented only 4.7 ± 1.6% of the CADM1^+^ cells and was compatible with cDC1. Finally, a third population with phenotype CD14^+^MHC-II^+^CD172a^+^ accounted for 39.6 ± 3.8% of the CADM1^+^ cells. No CD4^+^ cells (not shown) were detected, indicating that pDCs were probably not present in the Flt3L-derived cultures or in a very low proportion. Further phenotypical characterization (Figure 1B) showed that putative cDC2 were predominantly CD1^+^, CD163^*lo*^, DEC205^*lo*^, CD11R3^+^, CD11R1^+^, CD33^+^, and CD80/86^+^, while putative cDC1 were CD1^–^, CD163^–^, DEC205^+^, CD11R3^*lo*^, CD11R1^+^, CD33^+^, and CD80/86^+^. The CD14^+^ subset was predominantly CD1^+^, CD163^+^, DEC205^–^, CD11R3^+^, CD11R1^+^, CD33^–/*lo*^, and CD80/86^+^. The latter subset (CD14^+^) phenotypically resembled moDCs but could not be classified with absolute certitude due to the high expression of CADM1 (16). They will be designated as CD14^+^ DCs for the purpose of the present paper.

### Development Process of Flt3L-Derived Bone Marrow Cell Cultures

**FIGURE 2:**
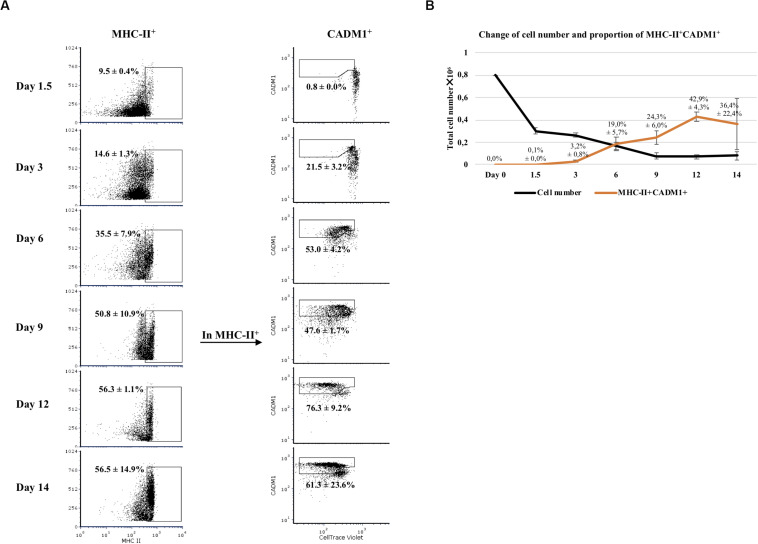
Development of Flt3-ligand-derived dendritic cells (Flt3L-DCs) during a 14-day culture period. **(A)** Bone marrow hematopoietic cells (BMHCs) were labeled with CellTrace Violet before stimulation with Flt3L. Cells were collected on days 1.5, 3, 6, 9, 12, and 14 and stained with antibodies anti-MHC-II and anti-CADM1. Proliferating cells (Violet^*lo*^) with a MHC-II^+^CADM1^+^ phenotype were considered as potential DCs. The gating strategy was MHC-II^+^CADM1^+^Violet^*lo*^. **(B)** Total number of viable cells in the culture versus time of incubation (black line), and proportion of potential DCs among viable cells versus time (orange line). Results were obtained with cells from three pigs. Average and standard deviation of the proportion of potential DCs are shown.

Next, we examined the development of CADM1^+^ and MHC-II^+^ cells in Flt3L-stimulated bone marrow cultures during the 14-day period (Figure 2). At day 0, no CADM1^+^ cells were detected in BMHC while MHC-II^+^ cells accounted for 16.0 ± 1.2% of the viable cells (data not shown). After 1.5 days of incubation, the number of viable cells dropped sharply (62.5 ± 3.5% of reduction; Figure 2B, black curve), suggesting that most of non-Flt3L-primed progenitors and other non-precursor cells died during this time. From that day on, CADM1^+^MHC-II^+^ cells started to develop. By day 6, an explosive increase of MHC-II^+^ cells was observed (35.5 ± 7.9% of total cells), among which 53.0 ± 4.2% were CADM1^+^Violet^*lo*^ (Figure 2A). This trend continued by day 12 with MHC-II^+^ reaching 56.3 ± 1.1% and CADM1^+^Violet^*lo*^ increasing to 76.3 ± 9.2% among MHC-II^+^ cells. A small decrease in the proportion of CADM1^+^ was observed by day 14 (Figure 2A). The development of CADM1^+^MHC-II^+^ cells determined the potential DCs among all viable cells that boosted at day 6 (19.0 ± 5.7% of viable cells) until day 12 (42.9 ± 4.3%) and finally declined slightly to 36.4% (Figure 2B, orange curve).

### Expression of Conserved DC Markers Assessed by RT-qPCR

**FIGURE 3:**
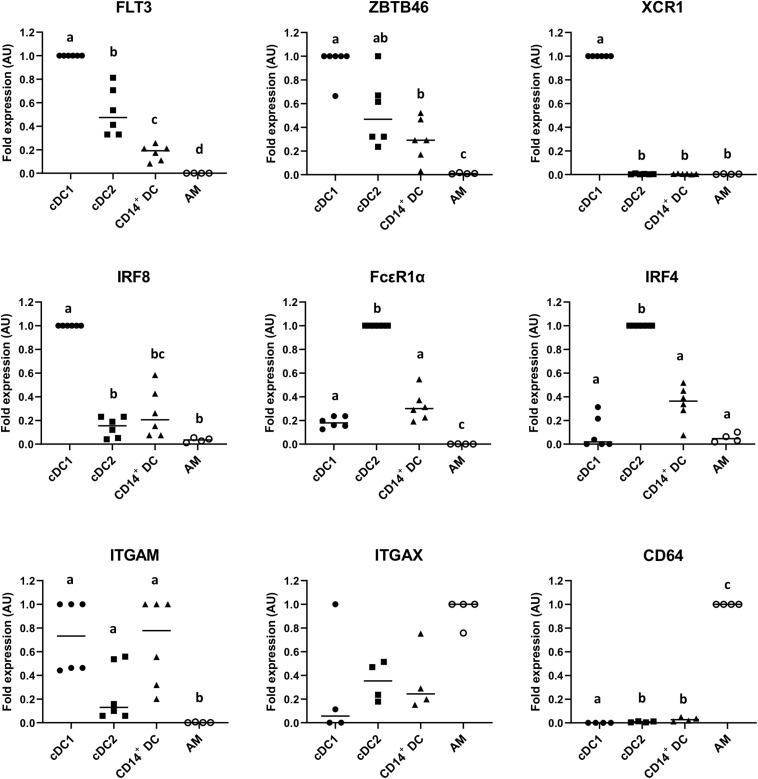
mRNA expression levels of FLT3, ZBTB46, XCR1, IRF8, FcεR1a, IRF4, ITGAM, ITGAX, and CD64 in sorted putative cDC1, cDC2, and CD14^+^ DCs were assessed by quantitative Real-Time PCR (RT-qPCR). Alveolar macrophages (AMs) were used as a different cell-type reference. For each gene, data were normalized to the expression of the reference gene *GAPDH* (glyceraldehyde 3-phosphate dehydrogenase) and shown as relative value [arbitrary units (AUs)]. For each animal, the cell population with the highest expression for each gene was considered as 1.0 and the other populations were normalized to it. Each symbol represents one animal; median is shown for each data set. Statistical significance was calculated by the Kruskal–Wallis test with multiple comparisons performed by Dwass–Steel–Critchlow–Fligner. Significant difference was indicated with different letters a, b, c,…; no letters were shown if no significance was found between groups.

To assess the expression of relevant canonical genes for the identification of DCs, the putative cDC1, cDC2, and CD14^+^ DC populations were sorted as indicated in Figure 1A, and MΦ were used for comparison. As shown in Figure 3, putative cDC1, cDC2, and CD14^+^ DCs expressed FLT3, with cDC1 showing the highest expression followed by cDC2 and CD14^+^ DCs; the same expression pattern occurred on ZBTB46, a transcription factor used to distinguish cDCs from other immune lineages in mice (27). Beyond that, putative cDC1 exclusively expressed XCR1 and IRF8; the assumed cDC2 overexpressed FcεR1a and IRF4, while CD14^+^ DCs showed low or intermediate levels of these genes. All three subsets expressed ITGAM. By contrast, AMs scarcely expressed these genes (FLT3, ZBTB46, XCR1, IRF8, FcεR1a, IRF4, and ITGAM), but overexpressed CD64 as expected. ITGAX (CD11c) was highly expressed on macrophages as well, but lower on the putative cDC1, cDC2, and CD14^+^ DCs. This is contrary to the result of pig blood cDCs (22) but somewhat consistent with the analysis of lung DCs (28).

**FIGURE 4:**
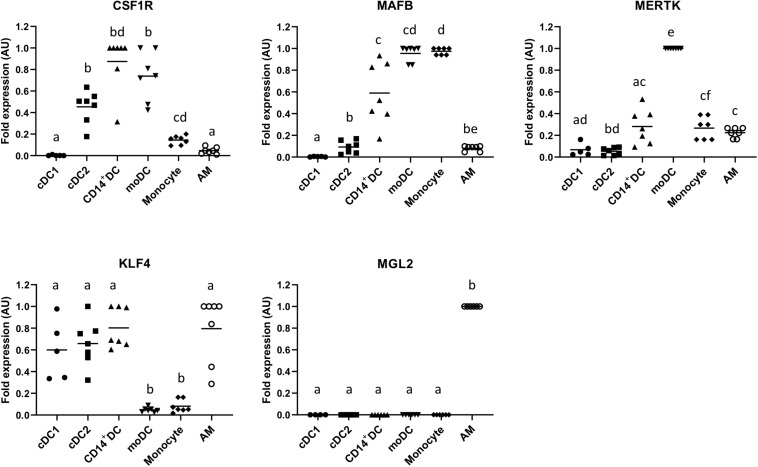
mRNA expression levels of CSF1R, MAFB, MERTK, KLF4, and MGL2 in sorted putative cDC1, cDC2, and CD14^+^ DCs were assessed by quantitative Real-Time PCR (RT-qPCR). Monocyte-derived DCs (moDCs), monocytes, and alveolar macrophages (AMs) were used as cell-type references. For each gene, data were normalized to the expression of the reference gene *GAPDH* (glyceraldehyde 3-phosphate dehydrogenase) and shown as relative value [arbitrary units (AUs)]. For each animal, the cell population with the highest expression for each gene was considered as 1.0, and the other populations were normalized to it. Each symbol represents one animal, median is shown for each data set. Statistical significance was calculated by the Kruskal–Wallis test with multiple comparisons performed by Dwass–Steel–Critchlow–Fligner. Significant difference was indicated with different letters a, b, c,…; no letters were shown if no significance was found between groups.

To further characterize the CD14^+^ subset, we assessed the expression of MAFB, CSF1R, MERTK, KLF4, and MLG2 on this cell population and compared them with cDC1, cDC2, monocytes, GM-CSF/IL-4 differentiated moDCs, and AMs. As shown in Figure 4, CD14^+^ DCs highly expressed CSF1R and MAFB and expressed MERTK at an intermediate level. They also expressed KLF4 but not MGL2.

### Functional Analysis of Putative cDC1, cDC2, and CD14^+^ DCs

**FIGURE 5:**
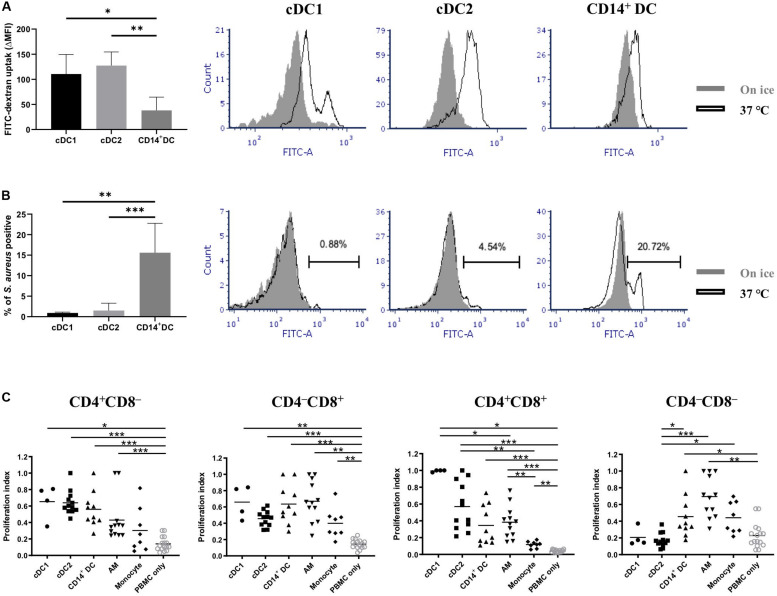
Assessment of antigen uptake and antigen presentation capabilities of putative cDC1, cDC2, and CD14^+^ DCs. **(A)** Endocytic capability of different DC populations was evaluated by incubating cells with dextran-FITC for 2.5 h either at 37°C or on ice with FITC fluorescence assessed by flow cytometry. Results are depicted as △median fluorescence intensity (ΔMFI) by subtracting MFI of cells incubated on ice from MFI of cells incubated at 37°C (data are the means of two pigs with each animal tested in triplicate). Overlapped histograms showed the fluorescence intensity of cells incubated at 37°C (open histograms) and cells incubated on ice (closed histograms). **(B)** Phagocytic activity of different DC populations was evaluated by incubating cells with Alexa 488-labeled inactivated *S. aureus* for 1 h either at 37°C or on ice and analyzed by flow cytometry. Results are depicted as cells positive for *S. aureus* (data are the means of four pigs with each animal tested in triplicate). Overlapped histograms showed the fluorescence intensity of cells incubated at 37°C (open histograms) and cells incubated on ice (closed histograms). **(C)** Sorted DCs (putative cDC1, cDC2, and CD14^+^ DC) and PBMCs (stained with CellTrace Violet) from allogeneic pigs were mixed at an APC:T cell ratio of 1:5 before being cultured for 5 days at 37°C. Alveolar macrophages (AMs) and monocytes were used as other cell-type references. Proliferation of CD4^+^CD8α^–^, CD4^–^ CD8α^+^, CD4^+^CD8α^+^, and CD4^–^ CD8α^–^ was assessed by CellTrace Violet dilution (see Supplementary Figure 2 for gating strategy). Each symbol corresponds to one type of DC reacting with one batch of PBMCs. The median is shown for each data set (horizontal bar). For each animal, the highest proliferation value was considered as 1.0, and the other populations were normalized to it. Data are from three independent experiments and a minimum of two animals for each cell type. Statistical significance was calculated by the Kruskal-Wallis test with multiple comparisons performed by Dwass-Steel-Critchlow-Fligner. ***p < 0.001, **p < 0.01, and *p < 0.05.

Two of the main properties of DCs are their capacities to capture antigens and then present them to activate naïve T cells. Thus, we tested the endocytic and phagocytic capabilities of the putative cDC1, cDC2, and CD14^+^ DCs. Dextran-FITC uptake and *S. aureus* phagocytosis experiments were set up for that purpose. All three populations presented an efficient uptake of dextran-FITC, with cDC1 and cDC2 showing higher capabilities than CD14^+^ DCs (Figure 5A). Of note, the histogram of cDC1 showed a bimodal distribution with two distinct subsets based on their uptake (high and moderate, Figure 5A). Although cDC1 contained cells of CD172a^–^ and CD172a^*lo*^, the two subsets with distinct uptake capabilities were not correlated with this molecule (Supplementary Figure 1) or with MHC-II (Supplementary Figure 1). cDC2 and CD14^+^ DCs showed a unimodal distribution. With regard to the phagocytosis of *S. aureus*, only a small fraction of cDC1 (0.9 ± 0.2%) and cDC2 (1.5 ± 1.8%) were phagocytic (Figure 5B); in contrast, CD14^+^ DCs showed a clear phagocytic activity (15.6 ± 7.2% of positive cells).

Next, we examined whether putative cDC1, cDC2, and CD14^+^ DCs possessed the capacity to activate allogeneic T cells in a mixed lymphocyte reaction (Figure 5C). The three DC populations did not show any significant difference in inducing CD4 or CD8 T cell proliferation. Of note, both the putative cDC1 and cDC2 were more efficient than CD14^+^ DCs or macrophages in inducing the proliferation of CD4^+^CD8^+^ T cells, with cDC1 being the most efficient type, although no significant difference was shown. Interestingly, cDC1 and cDC2 were less efficient in inducing proliferation of CD4^–^CD8^–^ T cells.

### Response to TLR Agonist Stimulation: Maturation Markers and Cytokine Patterns

**FIGURE 6:**
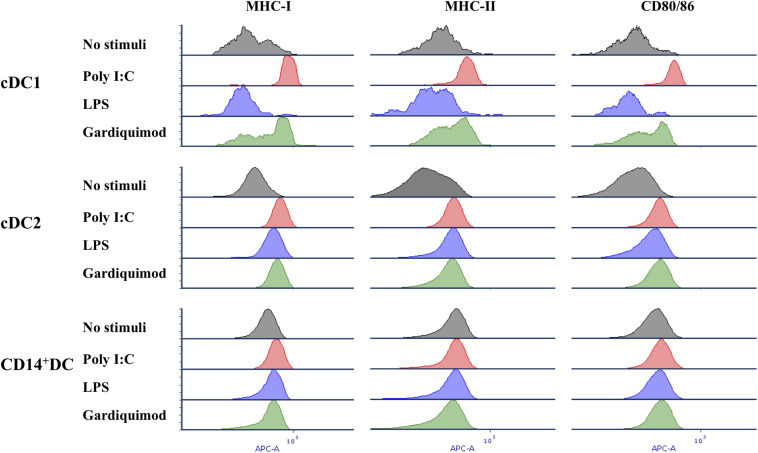
Expression of MHC-I, MHC-II, and CD80/86 on putative cDC1, cDC2, and CD14^+^ DCs after stimulation by TLR3, TLR4, and TLR7 agonists (10 μg/ml poly I:C, 1 μg/ml LPS, and 10 μg/ml gardiquimod, respectively) for 18 h or unstimulated as controls. Unstimulated control, gray histogram; poly I:C, red histogram; LPS, blue histogram; gardiquimod, green histogram. Data are representative of three pigs.

**FIGURE 7:**
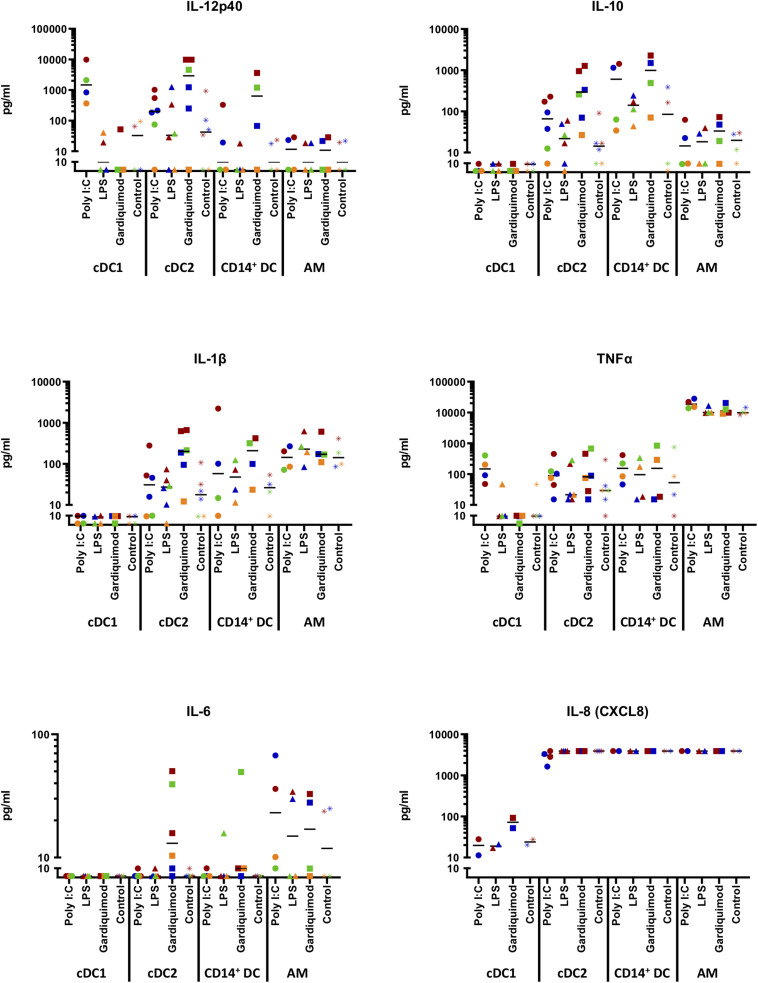
Cytokine production by sorted DC subsets (putative cDC1, cDC2, and CD14^+^ DCs) following TLR stimulation. Cells were stimulated for 18 h with 10 μg/ml poly I:C, 1 μg/ml LPS, 10 μg/ml gardiquimod, or left unstimulated as controls. The supernatants were harvested and tested for IFN-α, IFN-γ, IL-4, IL-6, IL-12p40, IL-10, IL-1β, TNF-α, and IL-8 (CXCL8) by multiplex immunoassay. Production of IL-12p40, IL-10, IL-1β, TNF-α, IL-6, and IL-8 (CXCL8) was shown, while IFN-α, IFN-γ, or IL-4 were not shown as rarely detected. Alveolar macrophages (AMs) were used as a different cell-type reference. The values were displayed with logarithmic *y*-axis. Symbols in different colors represent different animals. The median is shown for each data set.

Response of each DC subpopulation to TLR3, TLR4, and TLR7 ligands was assessed by determining the expression of MHC-I, MHC-II, and CD80/86 (Figure 6), and the cytokine profiles (Figure 7) after stimulation with poly I:C, LPS, and gardiquimod, respectively. The results showed that in cDC1, MHC-I, MHC-II, and CD80/86 were highly increased when stimulated by poly I:C or gardiquimod. Nevertheless, the cell viability was largely damaged by using gardiquimod or LPS, but not by poly I:C. In cDC2, MHC-I, MHC-II, and CD80/86 were increased by the addition of any of the three TLR ligands, but LPS was less effective in increasing MHC-I or CD80/86. On the contrary, CD14^+^ DCs had only a slight increase of MHC-I, but not MHC-II or CD80/86 when stimulated.

Regarding the cytokine patterns (Figure 7), no cytokines were boosted by LPS or gardiquimod in cDC1. IFN-α (<40 pg/ml), IFN-γ (<5 pg/ml), or IL-4 (<5 pg/ml) were rarely detected in any DC subset supernatant regardless of the TLR agonists used (data not shown).

Beyond that, the putative cDC1 produced substantial amounts of IL-12p40 (3284 ± 4429 pg/ml) when stimulated by poly I:C. Putative cDC2 and CD14^+^ DCs only produced IL12p40 in cells stimulated with gardiquimod (4292 ± 4599 pg/ml and 1218 ± 1686 pg/ml, respectively; Figure 7). AMs did not produce this cytokine.

With respect to IL-10, only putative cDC2 and CD14^+^ DCs produced this cytokine with CD14^+^ DCs making larger amount following the stimulation by gardiquimod (1083 ± 996.4 pg/ml) or poly I:C (666 ± 722 pg/ml), while cDC2 produced an intermediate amount (485 ± 509 pg/ml) in response to gardiquimod (Figure 7).

Regarding the pro-inflammatory cytokines (IL-1β and TNF-α), none was detected in the supernatant of cDC1 regardless of the TLR agonist used. In contrast, moderate amounts of IL-1β was detected in the supernatant of cDC2 (299 ± 277 pg/ml) or CD14^+^ DCs (215 ± 184 pg/ml) in both cases, through the TLR7-signaling pathway. Notably, while negligible TNF-α was produced by cDC2 or CD14^+^ DCs, AMs produced a considerable amount, regardless of the TLR ligand used (19,814 ± 6655 pg/ml with poly I:C; 11,514 ± 3305 pg/ml with LPS; and 12,984 ± 5169 pg/ml with gardiquimod; Figure 7).

In addition, a tiny amount of IL-6 was produced by cDC2 (20.5 ± 19.7 pg/ml) and CD14^+^ DCs (17.1 ± 22.0 pg/ml) upon gardiquimod treatment. IL-8 (CXCL8), an essential chemokine secreted from DCs involved in both Th1 and Th2 responses, was highly secreted by cDC2, CD14^+^ DCs, or AMs. It is noteworthy that there was a decrease in cDC2 cytokine secretion when these cells were stimulated by poly I:C, indicating that there might be an inhibition of IL-8 secretion by the activation of TLR3 signaling pathway. cDC1 did not produce this cytokine (Figure 7).

### Addition of SCF, GM-CSF, or IL-4 Does Not Improve the Production of cDCs

Once the cell populations originated after Flt3L stimulation were identified and characterized, we tried to optimize the production of DCs. Firstly, we compared the use of 20 ng/ml versus 100 ng/ml. This had no evident effect on the phenotype, the yield, or the relative proportions of different DC populations within the Flt3L-derived bone marrow cell cultures (not shown). Secondly, SCF was added to Flt3L-stimulated cultures at 20 ng/ml. The only noticeable effect was an increase in the yield of DC-compatible cells at the end of the 14-day incubation period that, on average, was 24% with no significant change in the type of cells produced or their relative proportions. Increase of SCF to 100 ng/ml did not show significant changes.

In the next step, GM-CSF was added to the bone marrow cultures after 6 days of the expansion of Flt3^+^ precursors, culturing for an extra 12 days (18-day period in total). The resulting cells on day 12 contained mostly (around 90%) CADM1^–^CD14^+^CD172a^+^, among which 30% were MHC-II^+^. These CD14^+^CD172a^+^ cells were further characterized as CD1^–^CD163^+^DEC205^+^. Nevertheless, by day 18, around 90% of CD163^+^ cells transformed to CD163^–^. Increasing of Flt3L up to 100 ng/ml did not substantially modify this profile (Supplementary Figure 3).

With the same scenario, addition of IL-4 produced mostly CADM1^–^CD14^+^CD172a^+^ cells with MHC-II^+^ increasing from 15% on day 12 to 30% by day 18. However, in this case, the CD14^+^CD172a^+^ population could be further subdivided based on the expression of CD1 (roughly a ratio of 1:0.8 of CD1^+^/CD1^–^) and CD163 (roughly a ratio of 1:1.5 of CD163^+^/CD163^–^). Only a small proportion of the CADM1^–^CD14^+^CD172a^+^ expressed DEC205 (Supplementary Figure 3).

## Discussion

The study of DCs and their interactions with pathogens is one of the main areas of research in immunology. One obstacle for these studies is the low proportion of DCs residing in tissues, which makes it difficult to obtain sufficient cell number for further analysis. Establishing an *in vitro* DC derivation model can make it more accessible for research i.e. the interaction of DCs with pathogens, the preliminary screening or testing hypotheses without need to perform costly whole-animal challenges. Here, we present a comprehensive characterization of cells produced in an Flt3L-based *in vitro* derivation method.

The use of Flt3L alone to stimulate BMHCs produced three main populations, which shared a CADM1^+^MHC-II^+^ phenotype and a variable expression of CD14 and CD172a. The first population was CADM1^+^CD14^–^MHC-II^+^CD172a^–/*lo*^, which presented full characteristics of cDC1: (1) it was further phenotyped as CD1^–^, CD163^–^, CD11R3^*lo*^, CD11R1^+^, and DEC205^+^, equal to the cDC1 described in porcine lymphoid tissues (15, 20), skin (21), lung (14), and blood (22). Of note, CD11b was positive on our cDC1, as revealed by the staining of CD11R3 and CD11R1 and the Quantitative Real-Time PCR (RT-qPCR) of ITGAM. This was compatible with the expression pattern of CD11b on blood cDC1 (22) but in contrast with the findings for lung cDC1 (14). In mice, CD11b is considered a specific marker of cDC2 (29); in pigs, according to the current result, CD11b expression on cDCs may depend on the cell type, as well as on the location of the cells, namely, blood or tissues; (2) in addition to FLT3 and ZBTB46, these cells highly expressed XCR1, which is a factor strictly associated with cDC1 (30) as well as IRF8, a driving factor of cDC1 development in human and mice (31); (3) they showed antigen presentation capability, particularly in activating allogenic CD8^+^CD4^+^ T cells, indicating the potential of inducing CD8^+^ effector/memory T cells; (4) they secreted a substantial amount of IL-12p40 following poly I:C stimulation, similarly to what was found in tonsil cDC1 (20) and, in lung cDC1 after influenza A virus infection (14). However, this differed from blood cDC1 where no IL-12p40 was detected by multiplex cytokine assay or qRT-PCR. It is worth noting that stimulation with poly I:C increased the viability of cDC1 in comparison to using other TLR ligands or just plain medium. Since poly I:C was a potent inducer of IL-12p40 in cDC1, it is tempting to speculate that this cytokine was the cause of the enhanced survival. Poor viability of cDC1 (32) and protection from cell death of cDC1 by IL-12 were previously reported (33, 34); (5) they were unresponsive to LPS or gardiquimod, compatible with the negligible expression of TLR4 and TRL7 on blood cDC1 (22); (6) they were incapable of producing IL-1β, same as human blood cDC1 where the scarcely expressed caspase-1 was reasoned (35); (7) they showed high endocytic capability. Interestingly, two subsets of cDC1 were identified in the dextran uptake experiments: low and high uptake, which did not correlate with CD172a or MHC-II expression levels; and (8) they showed very weak phagocytic activity, comparably to cDC2, but contrasting with the more efficient CD14^+^ DCs. This is in agreement with the finding in mice that the clearance of bulk particles was mostly by macrophages rather than cDCs (36). With respect to the yield, this population was the minor one in the Flt3L-derived cells, accounting for less than 5%. This fact suggests that either the precursors of cDC1 are scarce or the expansion of precursors and the survival of generated cDC1 require synergizing efforts from more factors. In human *in vitro* production models, cDC1 is also a minor population compared to cDC2 (17), which contrasts with the more balanced proportion of cDC1 and cDC2 in mice models (37).

The second, a major population, had a phenotype of CADM1^+^/CD14^–^/MHC-II^+^/CD172a^+^, representative of cDC2 (52% on average among CADM1^+^ cells). This population expressed a high level of FcεR1α, which has been identified as an overexpressed factor on cDC2 in human and pig tissues (14, 22, 38). IRF4, a driving factor of cDC2 differentiation (39), was also highly expressed. Combined with additional phenotypic markers CD1^+^, CD163^*lo*^, CD11R3^+^, and CD11R1^+^, this population was assigned to cDC2. However, the level of DEC205 on this population was lower than on the putative cDC1, which might be explained by our previous work in which expression of DEC205 was variable in cDCs obtained from different lymphoid tissues (15). When we extended the culture of these cDC2 for an additional 48 h, more cells gained DEC205 expression but lost CADM1 (data not shown), indicating that the lack of DEC205 expression might correspond to a relatively immature developmental stage. More interestingly, in our hands, the addition of different cytokines also had an impact on DEC205 expression. For example, addition of GM-CSF induced higher expression of DEC205. In other models, i.e., human DCs obtained *ex vivo* from skin, a similar variable pattern was observed for CD1a^+^ DDC (40). Furthermore, from a functional perspective, the putative cDC2 were weak in phagocytosing killed *S. aureus*, but efficient in endocytosing dextran, and were able to induce allogenic T cell proliferation. They produced IL-12p40 after TLR7 stimulation, which differed from cDC1 upon TLR3 stimulation. The secretion of IL-12p40 was not the specialty of one DC subtype in our model; this is contrary with what is reported in human (cDC2) (41) and mice (cDC1) (42). Moreover, the putative cDC2 were efficient in producing IL-10 or IL-1β, but not TNF-α, as was described in human cDC2 (43, 44). Another thing worth mentioning is the secretion of IL-6. Although it was a small amount (20.5 ± 19.7 pg/ml), it has been reported that, in mice, low amounts of IL-6 in the milieu can be *trans*-presented by the CD11b^+^Sirpα^+^ DCs to initiate the transcriptional program and generate pathogenic Th17 cells (45). Thus, the cDC2 produced in our study might play a guiding role in Th17 cell differentiation through secreting IL-6 in pigs. CD33 (Siglec-3) was positive on both cDC1 and cDC2, consistent with the study on human in which CD33 has a pan-distribution on myeloid pre-cDCs and cDCs (44). This is the first report of this molecule on pig DCs.

The third identified population was CADM1^+^CD14^+^MHC-II^+^CD172a^+^ (accounting for roughly 40% of the CADM1^+^ cells), designated as CD14^+^ DCs in this paper. It was further phenotyped as CD1^+^, CD163^+^, CD11R3^+^, CD11R1^+^, DEC205^–^, and CD33^–^. Based on the expression of FLT3, ZBTB46, FcεR1a, and IRF4 although low, they could be classified as cDCs. Furthermore, this CD14^+^ subset shared functional similarities with cDC2 in regard to T cell proliferation and cytokine production in response to TLR stimulation (secretion of IL-12p40, IL-10, and IL-6 upon gardiquimod treatment and very low or no TNF-α secretion following poly I:C or gardiquimod stimulation). This is in concordance with what was observed in CD14^+^ DCs in human interstitial dermis (46, 47). However, the relatively high expression of CSF1R, MAFB, and, to a lesser extent, MERTK would point these CD14^+^ cells toward a myelomonocytic origin. The CD14^+^ cells were also the ones with the highest phagocytic activity and were less responsive to TLR ligands in terms of maturation, elements favoring the monomyelocytic origin hypothesis. Nevertheless, moDC differentiation is boosted by GM-CSF rather than Flt3L (48, 49). Actually, in the present study, addition of GM-CSF radically changed the profile of cells obtained. Taken together, and considering that CADM1 is a cross-species, evolutionarily-conserved DC-specific marker that is not present in moDCs (38, 39), classification of the CD14^+^ subset as cDCs or moDCs cannot be entirely precise. In the work of Soldevila et al. (20), a similar CD14^+^ subset was also identified in porcine tonsils, which displayed an intermediate attribute between cDCs and moDCs in terms of phenotype, transcriptomics, and pathogen clearance. To note, morphologically cDC2 were produced as clusters of 5–15 cells, while CD14^+^ cells were seen as single cells, but both possessed typical DC dendritic and veiled extensions and lack of plastic adherence (data not shown). Other alternative hypotheses can also be formulated. The CD14^+^ cells might represent a subtype of cDC2, originated from the same precursors, but diversified into a distinct cDC2 subtype dependent on variable transcription clusters; even they could be in the transitional stage to cDC2. The presence of IRF4 and ZBTB46, and the comparable expression of KLF4 was in line with the two hypotheses.

CD4^+^ pDCs were not consistently detected in the cultures of our study, indicating that no or very few pDCs were present. According to Guzylack-Piriou et al. (19), less than 1% of Flt3L-stimulated cultures were compatible with pDC. This might be due to the lack of stromal cells in the culturing system (50), since their presence has proved to be indispensable for human pDC derivation *in vitro* (17).

The kinetic development of Flt3L-derived cells showed a sharp drop in cell numbers during the first days of incubation, suggesting death of non-stimulated Flt3^–^ cells in the culture; meanwhile, the proportion of MHC-II^+^ cells increased and afterward the CADM1^+^ population appeared and expanded. Based on this kinetic, we attempted to increase the yield of DCs by introducing SCF to induce hematopoietic progenitor expansion and subsequent survival (51). Although this resulted in a 24% increase in yield, the profile of cells produced was not affected. In contrast, the addition of GM-CSF at day 6 of incubation resulted in a proportion of CD14^+^ cells exceeding 90%, in agreement with what was previously observed in GM-CSF-derived bone marrow cultures (16, 19). Moreover, the produced cells were mostly CADM1^–^. These facts indicate that addition of GM-CSF may produce a different pattern of cDCs. In human cultures, omission of GM-CSF results in very low expression of DNGR-1^+^BDCA3^+^ DCs (cDC1 equivalent) (52), but in mice cultures, the lack of GM-CSF or its receptor did not result in a critical impairment (53). Another noticeable difference between several cytokine combinations is the expression of CD1. Most of the cells produced by Flt3L plus GM-CSF stimulation were CD1^–^. CD1 expression was restored by addition of IL-4. The combination of four factors (Flt3L, SCF, GM-CSF, and plus IL-4) generated a major population (>90%) with phenotype of CADM1^–^CD14^+^CD172a^+^, and a minor one (<10%) of CADM1^–^CD14^–^CD172a^+^. These cells were heterogeneous for CD1 and CD163 expression, with CD163 exclusively expressed by CD14^+^ cells (data not shown). Within CD1^+^ compartment, the CD14^–^CD163^–^ and CD14^+^CD163^+^ subsets may be analogous to, respectively, cDC2 and the recently identified human DC3 (54, 55). In our case, CD1 expression in Flt3L-stimulated BMHCs had a similar expansion kinetic to CADM1. The reasons why IL-4 restored that expression are not known yet.

Overall, the Flt3L-based *in vitro* derivation model produced *in vivo* equivalent cDC1 and cDC2 as well as a CD14^+^ population that, at present, cannot be precisely classified. This can be a suitable model to study the ontogeny of pig DCs, to assess potential functions of pig DCs as well as to study the interaction of DCs with porcine-related pathogens. The latter application permits *in vitro* examination of DCs in responding to pathogens without performing animal challenges, complying with the ethical obligation of reducing animal use in experimentation.

## Data Availability Statement

All datasets presented in this study are included in the article/Supplementary Material.

## Ethics Statement

The animal study was reviewed and approved by IRTA-CReSA Autonomous University of Barcelona.

## Author Contributions

YL contributed to the design and the execution of the whole panel of experiments, the analysis and the interpretation of the data, and the writing of the manuscript. EM contributed to the design of the experiments, the analysis and the interpretation of the data, and the writing of the manuscript. JH contributed to the design of the experiments and the interpretation of the data. LP-C contributed to the design and the execution of the phenotype characterization. ID assisted in the execution of part of the functional analysis. All authors reviewed the manuscript.

## Conflict of Interest

The authors declare that the research was conducted in the absence of any commercial or financial relationships that could be construed as a potential conflict of interest.
