# Supplementary material for: Development of Pig Conventional Dendritic Cells From Bone Marrow Hematopoietic Cells in vitro
Source: Front Immunol. 2020 Oct 8;11:553859. doi: 10.3389/fimmu.2020.553859 (PMC7580533; doi:10.3389/fimmu.2020.553859)
Supplement: Supplementary file 1 [file Presentation_1.PPTX]

## Slide 1
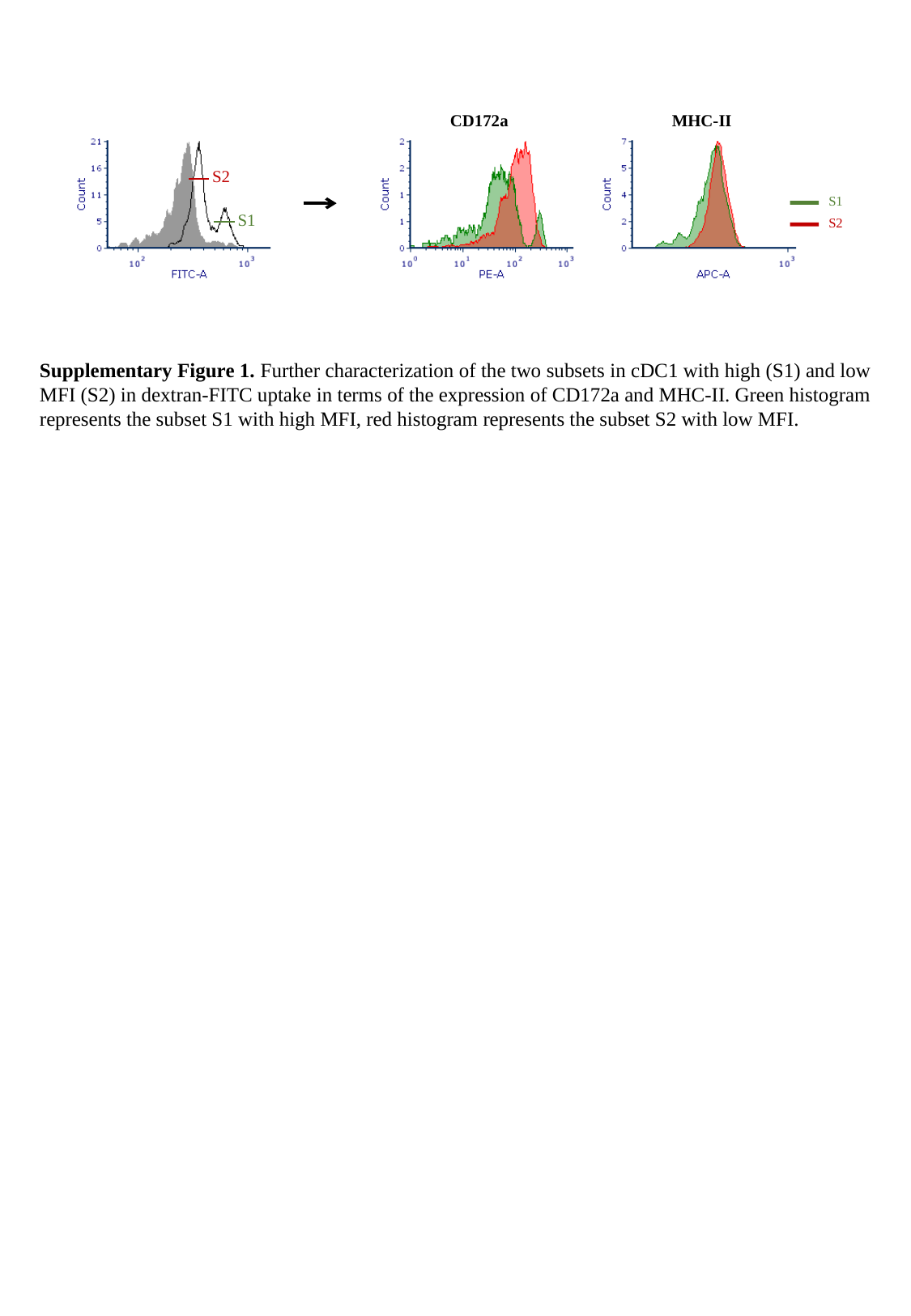

CD172a
MHC-II
S2
S1
S1
S2
Supplementary Figure 1. Further characterization of the two subsets in cDC1 with high (S1) and low MFI (S2) in dextran-FITC uptake in terms of the expression of CD172a and MHC-II. Green histogram represents the subset S1 with high MFI, red histogram represents the subset S2 with low MFI.
