# Supplementary material for: Development of Pig Conventional Dendritic Cells From Bone Marrow Hematopoietic Cells in vitro
Source: Front Immunol. 2020 Oct 8;11:553859. doi: 10.3389/fimmu.2020.553859 (PMC7580533; doi:10.3389/fimmu.2020.553859)
Supplement: Supplementary file 2 [file Presentation_2.PPTX]

## Slide 1
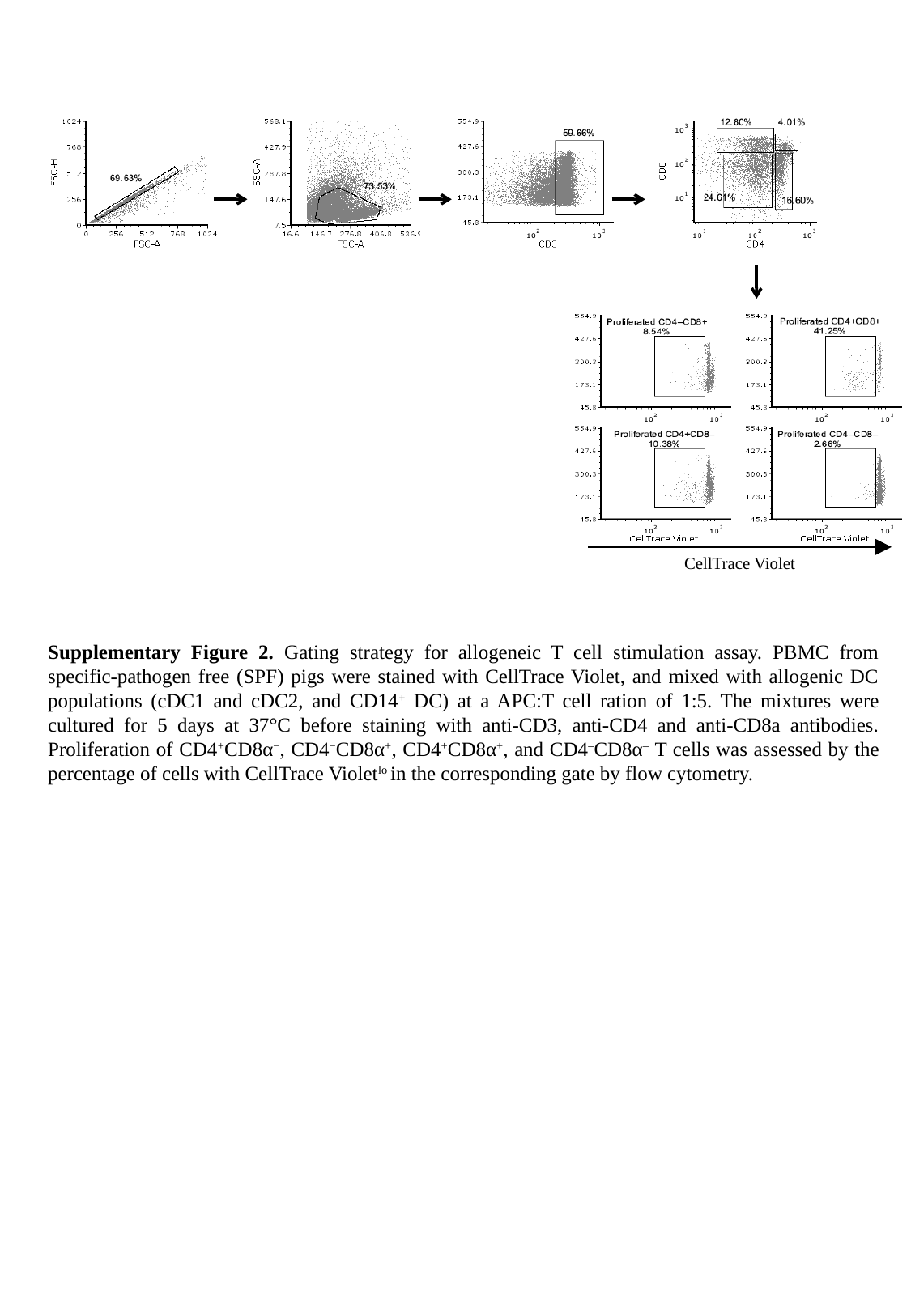

CellTrace Violet
Supplementary Figure 2. Gating strategy for allogeneic T cell stimulation assay. PBMC from specific-pathogen free (SPF) pigs were stained with CellTrace Violet, and mixed with allogenic DC populations (cDC1 and cDC2, and CD14+ DC) at a APC:T cell ration of 1:5. The mixtures were cultured for 5 days at 37°C before staining with anti-CD3, anti-CD4 and anti-CD8a antibodies. Proliferation of CD4+CD8α−, CD4−CD8α+, CD4+CD8α+, and CD4–CD8α– T cells was assessed by the percentage of cells with CellTrace Violetlo in the corresponding gate by flow cytometry.
