# Supplementary material for: Development of Pig Conventional Dendritic Cells From Bone Marrow Hematopoietic Cells in vitro
Source: Front Immunol. 2020 Oct 8;11:553859. doi: 10.3389/fimmu.2020.553859 (PMC7580533; doi:10.3389/fimmu.2020.553859)
Supplement: Supplementary file 3 [file Presentation_3.PPTX]

## Slide 1
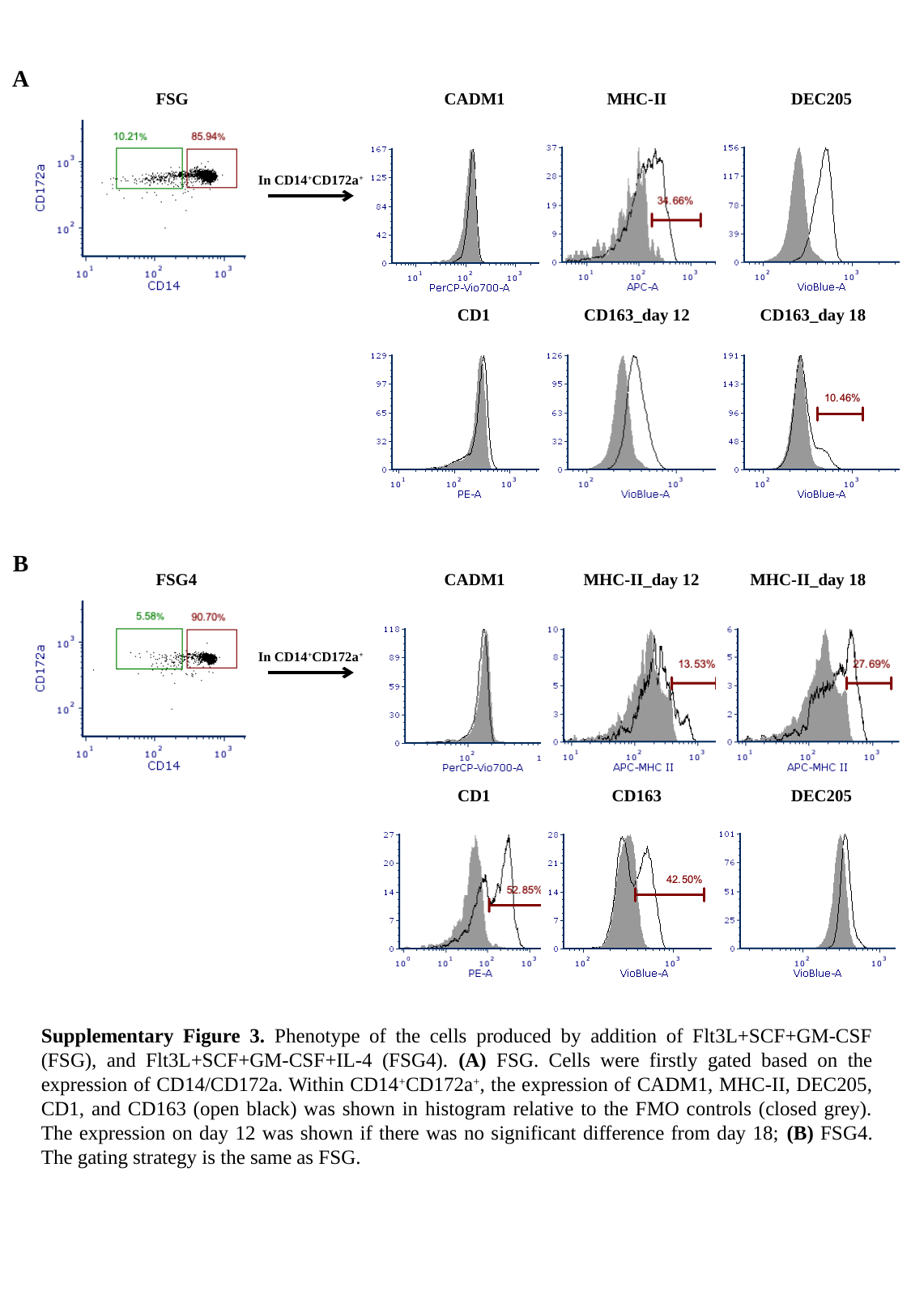

A
CADM1
MHC-II
DEC205
FSG
In CD14+CD172a+
CD1
CD163_day 12
CD163_day 18
B
CADM1
MHC-II_day 12
MHC-II_day 18
FSG4
In CD14+CD172a+
CD1
CD163
DEC205
Supplementary Figure 3. Phenotype of the cells produced by addition of Flt3L+SCF+GM-CSF (FSG), and Flt3L+SCF+GM-CSF+IL-4 (FSG4). (A) FSG. Cells were firstly gated based on the expression of CD14/CD172a. Within CD14+CD172a+, the expression of CADM1, MHC-II, DEC205, CD1, and CD163 (open black) was shown in histogram relative to the FMO controls (closed grey). The expression on day 12 was shown if there was no significant difference from day 18; (B) FSG4. The gating strategy is the same as FSG.
